# On the Origin of the Air between Multiple Tropopauses at Midlatitudes

**DOI:** 10.1100/2012/191028

**Published:** 2012-05-17

**Authors:** Juan Antonio Añel, Laura de la Torre, Luis Gimeno

**Affiliations:** ^1^Smith School of Enterprise and the Environment, University of Oxford, Oxford OX1 2BQ, UK; ^2^Ephyslab, Facultad de Ciencias de Ourense, Universidade de Vigo, 32004 Ourense, Spain

## Abstract

Multiple tropopauses are structures that regularly recur in the midlatitudes. Recent studies have relied on the notion of the excursion of tropical air from the upper troposphere into higher latitudes, thereby overlaying the tropopause of the midlatitudes. We herein analyse the origin and characteristics of the air at the Boulder radiosonde station, between the first and second tropopauses combining an analysis of radiosonde data with a Lagrangian approach based on the FlexPart model and ERA-40 analysis data. Our results show that the air between both tropopauses has its origin in midlatitudes.

## 1. Introduction

 Multiple tropopauses (MTs) are structures that regularly recur in the midlatitudes [1–3] and are extremely important for stratosphere-troposphere exchange (STE). Recent studies have relied on the notion of the excursion of tropical air from the upper troposphere towards higher latitudes, which then overlies the tropopause of the midlatitudes and is one of their main causes of MTs. Randel et al. [1] described the coincidence of double tropopause (DT) profiles with reduced amounts of ozone in the lower stratosphere (LS) with regions of increased transport from the tropics to higher latitudes above the subtropical jets. Pan et al. [4] suggested the association of DTs with intrusions of low-latitude air masses with low static stability and low ozone concentrations into the LS at midlatitudes, which they related to Rossby wave breaking events. However, Wang and Polvani [5] have recently suggested using an idealized model that air inside a DT structure comes from high latitudes. Vogel et al. [6] found that, in some cases, the contribution to mixing at levels between 330 K and 350 K is approximately equivalent to stratospheric and tropospheric intrusion.

 However, no clear understanding exists of the origin of MT phenomena, which could be associated with the overlapping of the tropical and extratropical tropopause, or to folding of the tropopause linked to the atmospheric circulation phenomena that characterise these latitudes, such as cut-off low (COL) systems [7], the movement of jet-streams, or baroclinic waves [8, 9]. Recent results show a widening of the Earth's tropical belt, which could further complicate this picture [10, 11].

 In order to address this gap in our understanding, we herein report on the origin of the air immediately above the first and below the second tropopause at the Boulder radiosonde station. We combine the data obtained from the water vapour soundings launched here with the results of reanalysis, based on Lagrangian analysis and fields of potential vorticity (PV).

 Our analysis relies on the fact that, if the origin of the MTs is related to excursions of tropical air over the extratropical tropopause, then we might expect there to be a contribution of air rich in moisture to the extratropical lowermost stratosphere. However, if the origin of the air is associated with the folding of the tropopause and with stratosphere-to-troposphere exchange, we would then expect to find air of stratospheric origin, that is, with a low moisture content and with higher concentrations of ozone.

## 2. Methods

 We herein consider data for the cold season in the Northern Hemisphere (November to March). The period of study was 1980–2009 for the computation of vertical profiles from soundings and 1980–2000 for the Lagrangian analysis, due to the availability of ERA-40 data.

 We chose the station of Boulder, Colorado (39.9°N, 105.3°W), for our study, mainly because it is a well-studied station and in a location likely to show the first tropopauses at tropical altitudes during summer and at extratropical altitudes during winter [2]. Sounding data were obtained from http://www.esrl.noaa.gov/gmd/ozwv/.

 The first and second tropopauses were computed for 319 different days for the analysis of vertical profiles and for 188 different days for the Lagrangian analysis. When there were days with two soundings at the same time, we chose the sounding that contained more values for water vapour. Tropopauses were defined according to the usual lapse-rate criterion given by the World Meteorological Organization [12] as follows.

- The first tropopause is defined as the lowest level at which the lapse rate decreases to 2°C/km or less, provided also the average lapse rate between this level and all higher levels within 2 km does not exceed 2°C/km.
- If above the first tropopause the average lapse rate between any level and all higher levels within 1 km exceeds 3°C/km then a second tropopause is defined by the same criterion as under (a). This tropopause may be either within or above the 1 km layer.

Next, we used the Lagrangian particle dispersion model FLEXPART developed by Stohl and James [13, 14], specifically v8.1. Runs were performed having in mind the complete longitudinal extension of the region between 10° and 65° North and with a vertical domain that spanned from sea level up to 22 km. The model was fed with ERA-40 reanalysis data [15]. Following the temporal resolution of this dataset, we used t_0_ to represent the time when a double tropopause (DT) was found in a sounding, obtaining results at 6-hour intervals beforehand. The maximum temporal domain for the computation of trajectories was 10 days. Longer computations were not considered to be relevant because 10 days is a typical residence time for water vapour in the atmosphere [16], during which we would expect to find a fingerprint of overlapping of the tropical tropopause. An analysis of the fields of PV was also undertaken, because this can be used to distinguish between tropospheric and stratospheric air masses [10]. The values are the ones given by FLEXPART for each particle.

 The density of particles was computed as the sum of the number of particles detected multiplied by the cosine of the latitude in order to weight the different latitudinal contributions. We integrated all the vertical levels in the latitude-longitude representation and all the latitudes in the altitude-longitude representation.

## 3. Results

**Figure 1:**
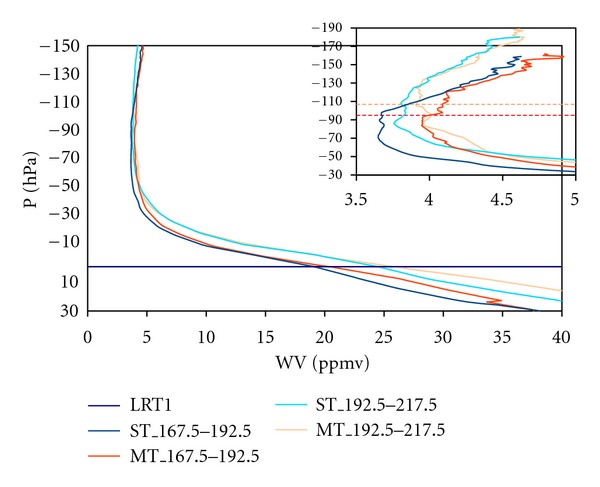
Vertical profiles of WV content (in parts per million by volume) relative to the pressure of the first tropopause (LRT1) shown for single (ST) and multiple tropopauses (MT) and over a range of pressures of occurrence (in hPa) of both phenomena. The part of the figure between 3.5 and 5 ppmv is enlarged in the upper right-hand corner in order to obtain a better insight of the changes in the vertical profile. The horizontal lines correspond to the second lapse-rate tropopause (colour denotes the pressure range where it happens).

 The computed vertical profiles of water vapour are shown in Figure 1 relative to the pressure of the first lapse-rate tropopause (LRT1). They are split into single (ST) and double tropopause (DT) cases and shown for two different vertical layers, namely, 167.5 hPa–192.5 hPa and 192.5 hPa–217.5 hPa. We split them thus because the soundings showed that they were layers in which the incidence of MTs was most common. Furthermore, it allowed us to check whether the MT events were lower or higher than these layers, being more or less representative of the layer between the MTs and the LS, respectively.

 It may be seen that, for ST events, the water vapour (WV) content immediately above the tropopause is lower than it is for DT events and that the WV content at LRT1 depends on the pressure of occurrence, independently of being a ST or MT event. It is also clear that the WV contents below the second lapse-rate tropopause (LRT2) are similar and independent of the pressure of occurrence. Moreover, the WV content just above LRT2 is greater for MT events with lower pressure values, suggesting the contribution of air masses rich in moisture, such as those from the tropics.

 This result appears to confirm the hypothesis of Pan et al. [4] of a tropical origin of the WV and air masses in MT events in subtropics and therefore support the view that MTs in the subtropics are a consequence of the overlapping of tropical tropopauses with extratropical ones. In order to obtain a better insight of this, we performed a Lagrangian analysis using FlexPart.

**Figure 2:**
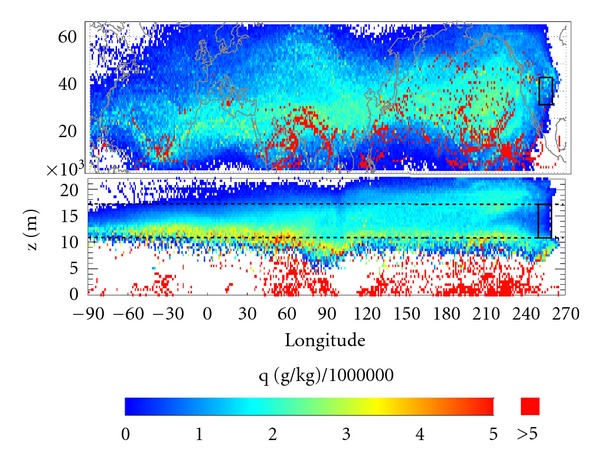
Specific humidity averaged for the ten days before time t_0_ (color scale). In the upper panel (latitude-longitude representation) the black square on the right-hand side represents the region near Boulder where particles arrive at t_0_. In the lower panels (height-longitude representation), the rectangle represents a boundary of 5 degrees of longitude surrounding the station at Boulder. The dotted black lines show the average heights of the first and second tropopauses.

 The Lagrangian analysis of average specific humidity *q* is shown in Figure 2. While it is not easy to see much of the detail, Figure 2 does show that maximum values occur for tropical and subtropical regions and for tropospheric levels.

**Figure 3:**
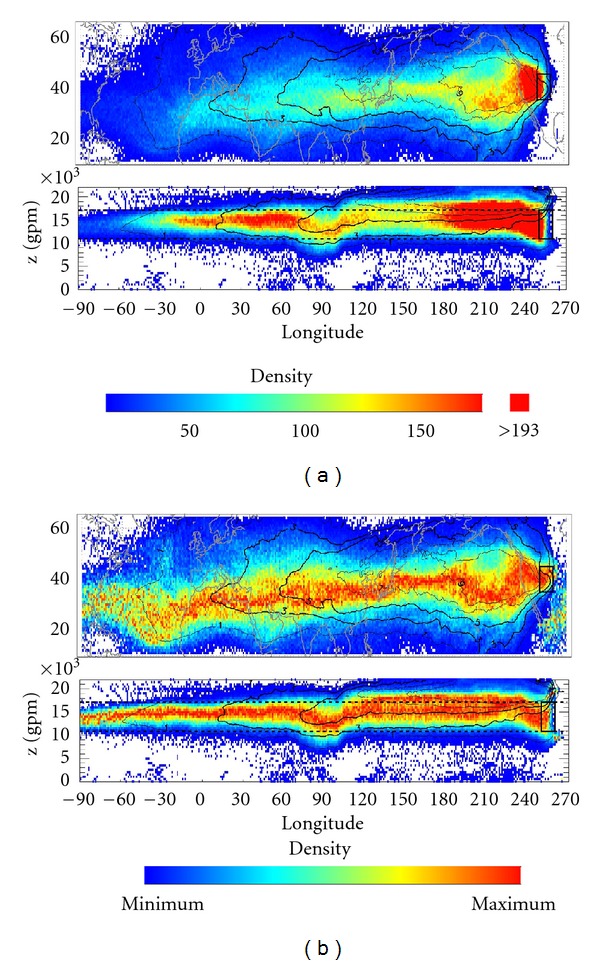
Particle density (colour scale) and smoothed contours of averaged PV (isolines of 1, 3, 5, 7, and 9 PVU). In the upper panels (latitude-longitude representation), the black square on the right-hand side represents the region near Boulder where particles arrive at t_0_. In the lower panels (height-longitude representation), the rectangle represents a boundary of 5 degrees of longitude surrounding Boulder. The dotted black lines show the average heights of the first and second tropopauses. (a) Sum for the ten days before t_0_. (b) Similar to (a) but representing the maximum density for each degree of longitude.

**Figure 4:**
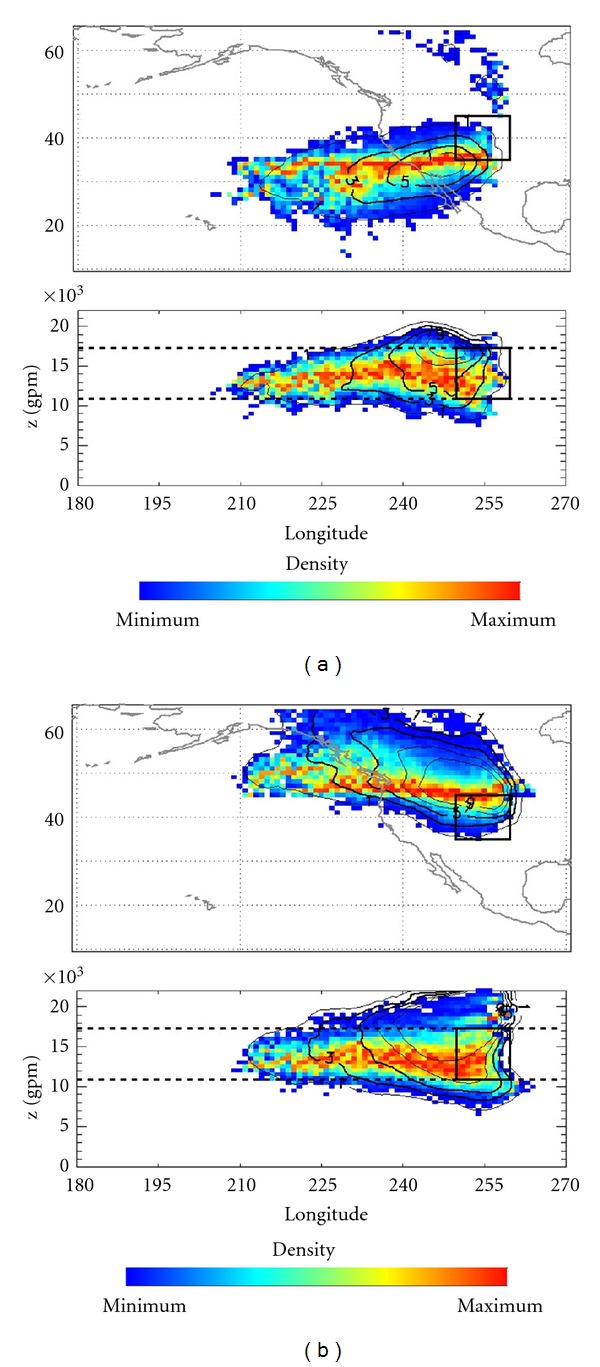
Similar to Figure 3(b) but (a) particles between the first and the second tropopauses whose origin 24 hours before t_0_ was at a latitude to the South of 35 degrees. (b) Similar to (a) but for particles with their origin at latitudes to the North of 45 degrees.

 Figures 3 and 4 show the density of the particles for the air masses over Boulder, respectively, 10 days and 24 hours before t_0_. The plots show the two different perspectives of latitude-longitude and altitude, with lines marking the average position of the first and second tropopauses for all the days when it was possible to compute it.

Figure 3 corresponds to the summed results for the particles between t_−240_ and t_−6_.  Figure 3(a), shows the absolute values. The latitude-longitude plot is less informative because the particles are concentrated around their destination. The use of the same colour scale for each longitude makes it difficult to see where the fastest particles originate. The height-longitude representation makes it possible to see how most of the particles that arrive at Boulder during MT events maintain their altitude for several days.

Figure 3(b) provides more information; this figure is similar to Figure 3(a) but the plot shows the particle density relative to each degree of longitude. It is thus possible to discern the source in terms of latitude and height for the maximum concentration of particles that arrive at Boulder. It is clear that air masses that contribute to the formation of MT events for the sum of the previous ten days are extratropical and that they have their source at similar altitudes to the MT event, above the first extratropical tropopause.

 In order to address more accurately the problem of identifying possible contributions of air masses with an extratropical or tropical origin, we performed the same analysis but for air masses whose source was at latitudes to the South of 35°N and to the North of 45°N. The results for the ten days beforehand are similar to those shown in Figure 3.  Figure 4 shows the results for t_−24_; from this figure, it is clear that particles arriving at Boulder, which were located below 35°N at any moment during the previous 24 hours, mostly stay at the same latitude and show values of PV greater than 1.5 or 2 PVU. Moreover, these particles usually lose altitude when approaching Boulder. This allows us to conclude that they could be stratospheric in origin and are probably associated with intrusions of stratospheric air in the troposphere, such as those associated with tropopausal folding. However, the effect of the orographic forcing of the Rocky Mountains is clear in the latitude-longitude plot of Figure 3(b). The particles arriving at Boulder that were located above 45°N at any moment in the previous 24 hours maintain the same latitude and altitude and increase their PV, suggesting fast mixing with air masses from upper levels with lower temperatures.

## 4. Concluding Remarks

 Previous literature on the causes of MTs in extratropical regions is somewhat contradictory, either attributing them to excursions of the tropical over the extratropical tropopause, or to STEs associated with synoptic atmospheric phenomena.

 From our results, it is clear that residence times for air masses between the first and second tropopauses are no longer than 24 hours. Moreover, the source of these air masses is mainly stratospheric, as may be concluded from the associated high values of PV (>3 PVU in most cases). It may also be seen that the values of PV associated with these air masses are inversely proportional to their distance from the Boulder station. This could be evidence for a link with a cyclonic circulation at upper levels (local maximum of PV) that is characteristic of COLs. Sometimes the values of PV increase near Boulder and then decrease, which might possibly be an indication of tropopausal folding.

 Air masses with tropospheric characteristics are only present in a few cases and when considering periods longer than ten days, which is in agreement with the findings of Vogel et al. [6]. This result should not come as a surprise, however, because meridional circulation patterns increase in importance at longer periods of time. In the same way, given these residence times, it is improbable that the transport of water vapour from lower latitudes towards the extratropical lowermost part of the stratosphere is a main cause of MTs; our findings indicate that it is rather more likely that there is a relationship with the radiative effect of O_3_ transported from upper levels to the upper troposphere/lower stratosphere.

 In conclusion, our findings provide one additional source of information for the discussion of the origin of MT events at midlatitudes, suggesting that extratropical phenomena associated with exchanges between the stratosphere and the troposphere are in most cases a much more probable cause than overlapping with the tropical tropopause.
